# Ischemia modified albumin as a useful marker for diagnoses and management of diabetic retinopathy

**DOI:** 10.12669/pjms.38.4.4813

**Published:** 2022

**Authors:** Winay Kumar, Russell Seth Martins, Nargis Anjum, Syeda Sadia Fatima

**Affiliations:** 1 Dr. Winay Kumar, MBBS, MPhil. Department of Physiology, Jhalawan Medical College Khuzdar, Aga Khan University, Karachi, Pakistan; 2 Russell Seth Martins, MBBS Student. 4^th^ Year Student, Medical College, Pakistan; 3 Dr. Nargis Anjum, MBBS, MPhil. Department of Physiology, Karachi, Medical and Dental College, and University of Karachi; 4 Dr. Syeda Sadia Fatima, MBBS, MPhil, PhD, FHEA (UK). Department of Biological and Biomedical Sciences, Aga Khan University, Karachi, Pakistan

**Keywords:** Diabetes retinopathy, Diabetes, Ischemia modified Albumin, Intravitreal anti-vascular endothelial growth factor

## Abstract

**Objectives:**

Ischemia modified albumin (IMA) may aid in the early detection and management of diabetic retinopathy (DR). In this study, we examined the relationship between IMA and DR, and the effect of intravitreal anti-vascular endothelial growth factor (anti-VEGF) on IMA levels in patients with DR.

**Methods:**

This Quasi-experimental study was conducted from March-December 2018 at a Al-Ibrahim Eye Hospital in Karachi, Pakistan. Adult patients (age ≥ 18 year) with Type-2 diabetes mellitus (T2DM) presenting to the Diabetic Clinic were categorized as control (Group-A n=30: DM without DR) or case (Group-B n=59: DM with DR). Patients in Group-B received an intravitreal injection of bevacizumab (anti-VEGF). Visual acuity, retinoscopy and serum IMA were recorded at baseline and at a 30-day follow-up for both groups.

**Results:**

A significant drop in IMA levels was seen one month after bevacizumab (IMA baseline: 1590.82±121.22 and follow up: 940.8±91.26; p<0.01) in Group-B subjects. Visual acuity (VA) of patient in Group-B also improved one month after bevacizumab injection in both eyes (p<0.001). Whereas, the IMA levels in Group-A showed an upward rising trend after one month (baseline 448.80±22.4ng/ml and follow up 522.21±33.15 ng/ml; p>0.05) indicating disease progression.

**Conclusion:**

Ischemia modified albumin may be used as an effective and novel screening biomarker for assessing oxidative stress associated with DR, and to quantify response to and prognosis after intravitreal bevacizumab injection for DR.

## INTRODUCTION

Worldwide, millions of individuals lose their vision because of diabetic retinopathy (DR).^1^ Amongst diabetics globally, the prevalence of DR is around 27%.^2^ In South Asia, the prevalence of DR amongst diabetics ranges from 14.5-18% in India^3^ to around 27-73.1% in Pakistan, with variability attributed to differences in sampling locations.^4^-^6^ Fifty seven percent cases of visual loss can be prevented by earlier diagnosis and by apropriate treatment.^7^

Diabetic retinopathy may be categorized as non-proliferative diabetic retinopathy (NPDR) or proliferative diabetic retinopathy (PDR). The main feature of PDR is retinal neovascularization due to ischemia,^8^ which is mediated by the release of vascular endothelial growth factor (VEGF). However, these newly formed vessels are immature and fairly brittle, resulting in complications such as vitreous hemorrhage, retinal detachment and loss of vision.^9^

Ischemia modified albumin (IMA) has emerged as a novel and inexpensive biomarker for assessing ischemia and oxidative stress in diabetic retinopathy.^9^,^10^ IMA is a structurally modified form of albumin that results from ischemia and the consequent oxidative stress (OXS) and free radicals. As the symptoms of DR are not apparent earlier in the disease process, IMA may help in the early detection of ischemia and subsequent management.^11^

Newer treatment options for PDR and clinically significant diabetic macular edema (DME) include intravitreal anti-vascular endothelial growth factor (anti-VEGF). A remarkable regression of retinal neovascularization is evident after one-month of treatment with intravitreal anti-VEGF, in addition to improvement in visual acuity.^12^ Thus, serum IMA levels may help guide the treatment efficiency and dosages of intravitreal anti-VEGF.^13^ In this study, we examined the relationship between IMA and DR, and the effect of intravitreal anti-VEGF on IMA levels in patients with DR.

## METHODS

This quasi-experimental study was conducted from March 2018 to December 2018 at the Al-Ibrahim Eye Hospital, in collaboration with the Department of Biological and Biomedical Sciences, Aga Khan University, and the Department of Physiology, Basic Medical Sciences Institute, Jinnah Post Graduate Medical Centre, Pakistan. The study was approved by the ethics review committee at the Jinnah Post Graduate Medical Centre (No.F.2-81-IRB/2018-GENL/4962/JPMC). Non-probability consecutive sampling was used to approach patients with Type-2 diabetes mellitus (T2DM) presenting to the Diabetic Clinic. Exclusion criteria included any previous history or evidence of myocardial ischemia, diabetes nephropathy, deep vein thrombosis, stroke, lower extremity ischemia, acute infectious disease, chronic inflammatory disease, cancer, retinal disease due to other causes (e.g., vitreomacular traction), previous scattered pan-retinal photocoagulation treatment, or significant cataract (which does not allow complete ocular examination and proposed measurements).

A verbal and written informed consent were taken from all subjects. They were categorized as control (Group-A n=30: DM without DR) or case (Group-B n=59: DM with DR) (DR as diagnosed according to the International Clinical Diabetic Retinopathy Disease Severity Scale).^14^

Patients’ visual Acuity (VA) was measured by using Snellen chart. Ophthalmological examination was performed by an ophthalmologist using slit lamp biomicroscopy, and the dilated fundus was examined using a + 90 dioptre lens and indirect ophthalmoscopy. Presence of macular edema or PDR was noted. All patients were re-examined after one month of their initial evaluation. Blood samples were collected in the morning, after an overnight fast, between 10.30 to 11.30 am at the clinic. Serum was separated and stored at -80°C till further processing. Fasting blood glucose (glucose oxidase-phenol-aminophenazone method; Merck, France), lipid profile (enzymatic endpoint method) were measured. Ischemia modified albumin was measured using an ELISA kit (USCN Life Science Inc., USA; ELISA Kit, Cat. No: E90825H). For Group-B (patients with DR) IMA levels were assessed at baseline before they received an intraocular injection of anti-VEGF and at one-month interval after the anti-VEGF injection. In preparation for bevacizumab (anti-VEGF agent) injection, local anaesthetic eye drops (Alcaine – Alcon, Belgium) were used, pupils were dilated with tropicamide (Mydriacyl 1% - Alcon, Belgium), and eyes were prepared using 5% povidone-iodine. For stabilization of eyelids, an eyelid speculum was used. Bevacizumab injection (Avastin, Genentech Inc. South San Francisco, CA, USA) 1.25 mg (0.05 ml) was injected at 3.5 to 4mm posterior to the limbus through the inferiotemporal pars plana with a tuberculin syringe and 30-gauge needle. After injection, the perfusion of retinal artery was checked by indirect ophthalmoscope. One drop of Vigamox (Alcon, Belgium) antibiotic was instilled. Patients were advised to use topical antibiotics for seven days.^15^

Data analysis was performed using IBM SPSS Statistics (version 21; SPSS Inc., Chicago, IL, USA). Data on continuous variables i.e. biophysical (age, BMI, visual acuity etc.) and biochemical parameters (lipid profile, fasting blood glucose, IMA etc.), were described using mean and standard deviation (SD). Categorical variables were presented as frequencies and percentages. Statistical comparisons were performed by student t-test and Man Whitney-U-Test for continuous/quantitative variables, and Chi square or Fischer exact test for categorical variables. Paired sample t-test was used to compare mean serum IMA and visual activities at baseline with those at the one-month follow-up. For all analyses, a p-value < 0.05 was considered significant.

## RESULTS

**Fig.1:**
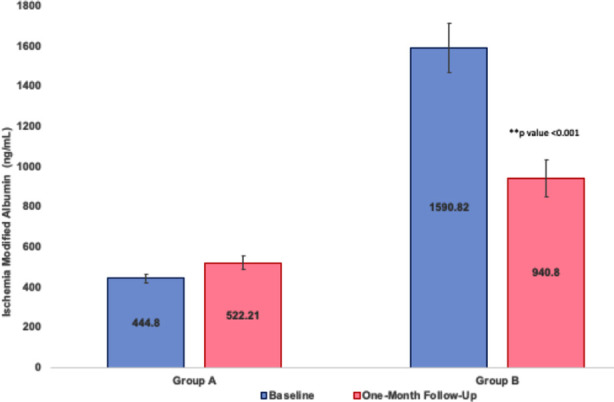
IMA levels before and after giving treatment. A significant decrease in levels was observed in follow-up cases. Values expressed as Mean ± SD. Comparison from baseline to follow-up was made using paired sample t-test. **Statistically significant as compared to compared p<0.01

A total of 89 patients with T2DM, between the age ranging from 30-70 years, were included in this study. No significant differences in the age, gender ratio, or BMI between Group-A and Group-B (Table-I) were seen. Group-B had a significantly higher LDL-cholesterol, triglycerides and HbA_1C_, and significantly lower HDL-cholesterol, than Group-A (Table-II). Fig.1 shows the baseline and follow up Ischemia modified albumin (IMA) levels; a significant drop in the IMA levels was seen after the first treatment that depicts a lowering of the inflammatory state and improving the retinal blood flow (Group-B IMA baseline: 1590.82 ± 121.22 and follow up: 940.8 ± 91.26; p<0.01) in comparison to Group-A (baseline 448.80 ± 22.4ng/ml and follow up 522.21 ± 33.15 ng/ml; p>0.05). This finding was corroborated by the improvement in visual acuity of these patients post treatment (p<0.01) (Table-III).

**Table-I:** Participants’ Baseline Biophysical Data.

		Group-A (DM without DR) (n=30) Mean ± SD	Group-B (DM with DR) (n=59) Mean ± SD	P value
Age (year)		42.70 ± 8.90	55.94 ± 8.08	0.588
Weight (kg)		69.40 ±14.26	66.28 ± 11.33	0.138
Body Mass Index (kg/m^2^)		25.12 ± 4.61	24.74 ± 3.66	0.694
Gender	Male	18 (60.00)	35 (59.32)	0.695
	Female	12 (40.00)	24 (40.67)	
Systolic Blood Pressure (mmHg)		132.50 ± 16.50	145.79 ± 20.98	0.024
Diastolic Blood Pressure (mmHg)		82.00 ± 10.05	93.07 ± 6.94	0.022

**Table-II:** Baseline Biochemical Parameters of the study Cohort.

	Group-A (DM without DR) (n=30) Mean ± SD	Group-B (DM with DR) (n=59) Mean ± SD	P value
Fasting Blood Glucose (mg/dl)	129.25 ± 25.23	121.97 ± 24.92	0.052
Random Blood Glucose (mg/dl)	214.95 ± 82.33	224.35 ± 42.38	0.524
Low Density Lipoprotein Cholesterol (mg/dl)	83.10 ± 4.83	163.56 ± 12.74	0.001
High Density Lipoprotein Cholesterol (mg/dl)	42.15 ± 4.49	36.89 ± 7.05	0.044
Cholesterol (mg/dl)	153.90 ± 17.29	250.76 ± 45.44	0.001
Triglyceride (mg/dl)	109.40 ± 14.88	158.05 ± 19.91	0.035
HbA1c (%)	6.12 ± 0.72	8.90 ± 1.02	0.015
Serum Creatinine (mg/dl)	0.69 ± 0.25	0.92 ± 0.38	0.588

**Table-III:** Effect of anti-VEGF treatment on Visual Acuity and DR in Group-B.

	Baseline Visual Acuity (Mean ± SD)	Follow-Up Visual Acuity (Mean ± SD)	P value
Right Eye	20/116 ± 10	20/67± 5	<0.001
Left Eye	20/120 ± 10	20/74 ± 6	<0.001
	Baseline Fundoscopy n (%)	Follow up Fundoscopy n (%)	
Macular Edema	27 (45.8)	Not applicable	
Proliferative Diabetic Retinopathy	12 (20.3)		

^^^On follow-up either the degree of macular edema was reduced, or the signs and symptoms of DR were improved. All patients were still classified as DR till the treatment was completed as per protocol.

## DISCUSSION

In this study, we assessed IMA levels in DM patients with and without DR, and reported the effect of intravitreal anti-VEGF on IMA levels in patients with DR. Our findings revealed significantly higher levels of IMA in DM patients with DR as compared to patients without DR, and a substantial reduction in IMA and improvement in visual acuity at one-month post-intravitreal anti-VEGF injection in patients with DR.

IMA has been described as a biomarker of general microvascular injury in DM, with non-significant differences seen between the various diabetic vascular complications.^16^ Nevertheless, previous studies corroborate the utility of IMA in assessing risk for DR. In India, Reddy et al. reported significantly higher IMA levels in patients with DR, as compared to health controls.^17^ Moreover, a study by Turk et al. demonstrated a high sensitivity of IMA towards DR, with an area under the receiver operating curve of 0.789.^9^ A subsequent study by Kirboga et al. from Turkey also showed increased serum IMA in patients with DM with DR, as compared to healthy controls.

While intravitreal anti-VEGF has a well-established role in the treatment of proliferative DR, agents such as aflibercept have also shown promise in the reversal of nonproliferative DR.^18^ A study by Soiberman et al. in Israel^13^ demonstrated how intravitreal anti-VEGF resulted in lower serum IMA levels in a sample of patients with retinal vascular disease due to age-related macular degeneration or diabetic macular edema. However, to the best of our knowledge, our study is the first to demonstrate the lowering effect of intravitreal anti-VEGF injection on serum IMA levels in patients with DR. The role of anti-VEGF in DR is based on its inhibition of the pathologic neovascularization driven by higher levels of VEGF, which are induced by retinal hypoxia.^19^ Our findings of lower IMA levels after anti-VEGF treatment are somewhat contradictory to available literature, which show reduced retinal perfusion mediated by vasoconstriction caused by anti-VEGF agents, and hence exacerbation of hypoxia and increased IMA.^20^ However, anti-VEGF treatment has been shown to reduce inflammation and chemotaxis in the retina,^21^ which could in turn lower IMA due to decreased oxidative stress.

Visual acuity (VA) of patient in Group-B improved one month after bevacizumab injection. Abbasi et al. reported that Bevacizumab is extremely useful in improving visual acuity in patients of DME and fresh vitreous hemorrhage as it accelerates resolution of hemorrhage.^22^ Intravitreal injection of Bevacizumab is improving VA in patients with PDR, with the effect most prominent in the patients with T2DM for more than 10 years. This was demonstrated in a study by Bahoo et al. in 2011.^23^

The use of control group comprising of patients with DM but without DR serves to partially negate the confounding effect of other diabetic vascular complications on serum IMA levels. However, baseline differences between the two groups in our study may represent potential confounding factors. These included higher blood pressure, a worse lipid profile, and higher HbA_1C_ in the group with DR, as compared to the control group. Nevertheless, to the best of our knowledge, our study is the first to compare the serum IMA of patients with DR to that of patients with DM without evidence of DR.

## CONCLUSION

Ischemia modified albumin may be used as an effective and novel screening biomarker for assessing oxidative stress associated with DR, and to quantify response to and prognosis after intravitreal bevacizumab injection for DR.

### Authors’ Contribution:

**SSF:** Conceived, designed and did statistical analysis, editing of manuscript and is responsible for integrity of the study.

**WK:** Performed data collection and manuscript writing.

**RSM & NA:** Prepared the manuscript.

All authors did review and final approval of manuscript.
